# Sloths like it hot: ambient temperature modulates food intake in the brown-throated sloth (*Bradypus variegatu*s)

**DOI:** 10.7717/peerj.875

**Published:** 2015-04-02

**Authors:** Rebecca N. Cliffe, Ryan J. Haupt, Judy A. Avey-Arroyo, Rory P. Wilson

**Affiliations:** 1 Swansea Lab for Animal Movement, Biosciences, College of Science, Swansea University, Swansea, Wales, United Kingdom; 2 The Sloth Sanctuary of Costa Rica, Limon, Costa Rica, Central America; 3 Department of Geology and Geophysics, University of Wyoming, Laramie, WY, United States

**Keywords:** Sloth, Metabolism, Digestion, Thermoregulation, *Bradypus*

## Abstract

Sloths are considered to have one of the lowest mass-specific metabolic rates of any mammal and, in tandem with a slow digestive rate, have been theorized to have correspondingly low rates of ingestion. Here, we show in a study conducted over five months, that three captive *Bradypus variegatus* (Brown-throated sloths) had a remarkably low mean food intake of 17 g kg^−1^day^−1^ (SD 4.2). Food consumption was significantly affected by ambient temperature, with increased intake at higher temperatures. We suggest that the known fluctuation of sloth core body temperature with ambient temperature affects the rate at which gut fauna process digesta, allowing for increased rates of fermentation at higher temperatures. Since *Bradypus* sloths maintain a constantly full stomach, faster rates of fermentation should enhance digestive throughput, increasing the capacity for higher levels of food intake, thereby allowing increased energy acquisition at higher ambient temperatures. This contrasts with other mammals, which tend to show increased levels of food intake in colder conditions, and points to the importance of temperature in regulating all aspects of energy use in sloths.

## Introduction

To balance energy intake with expenditure (Emlen, 1966; MacArthur & Pianka, 1966), animals have to deal with food intake being modulated by internal factors, such as physiological state (Mertens, 1994), and external factors, such as variation in the environment (Brobeck, 1948). For endothermic mammals, one critical parameter is ambient temperature because, as this drops below the thermo-neutral zone, the increased loss of energy due to thermoregulatory demands has to be counteracted by increased intake, i.e., if it gets cold most mammals tend to eat more (e.g., Donhoffer & Vonotzky, 1947). Sloths deviate from this general mammalian plan though, having a reduced ability to maintain body temperature, probably to save energy (Irving, Scholander & Grinnell, 1942; McNab, 1978; Montgomery & Sunquist, 1978). Similar to many poikilotherms, they rely on behavioural methods of thermoregulation, exhibiting daily fluctuations in core temperature of up to 10 °C (Britton & Atkinson, 1938; Irving, Scholander & Grinnell, 1942; Montgomery & Sunquist, 1978). This fluctuation is in stark contrast to most endothermic mammals, which maintain a constant core temperature of approximately 36 °C regardless of the ambient temperature (Phuoc & Ngoan, 2005), and raises the question of how sloths might balance food intake with temperature. Indeed, there is a chance that even the thermic effect of food digestion (Brobeck, 1948) might impact their heat balance.

In addition to a low and variable body temperature, sloths have one of the lowest metabolic rates amongst mammals (McNab, 1982; Nagy & Montgomery, 1980). While the true metabolic rate for *B. variegatus* is unknown, reported rates vary anywhere from 40–74% of the predicted value given for their body mass (Irving, Scholander & Grinnell, 1942; Brody, 1945; Nagy & Montgomery, 1980; McNab, 1982). As a result of this low metabolic rate, *B. variegatus* sloths are able to subsist on an extremely low-energy diet, feeding predominantly on leaves with a notably low caloric content (Chiarello, 1998; Coley & Barone, 1996) and measurable toxicity due to the presence of alkaloids, phenols and terpenes (Freeland & Janzen, 1974; Whittaker & Feeny, 1971). Although a wide variety of plant species are known to constitute the diet of *Bradypus* sloths, those with low toxin content such as *Cecropia sp*. are often favoured, and as with many other arboreal folivores, younger leaves are always preferred due to the lower tannin and fibre content aiding digestibility (Chiarello, 1998; Reid, 1997). It has recently been suggested that *Bradypus* sloths will supplement this low calorie diet by consuming the lipid-rich algae that is present on the fur (Pauli et al., 2014), although the extent of the nutritional benefits and the frequency of this behaviour are unknown.

In general, arboreal folivores compensate for a low-calorie diet by consuming relatively large quantities of food. For example, sympatric mantled howler monkeys (*Alouatta palliata*) consume three times as many leaves per kilogram (kg) of body mass as sloths (Nagy & Milton, 1979). This option appears untenable for the sloth, despite having an extremely large stomach with abdominal contents accounting for up to 37% of their ∼4.5 kg body mass (Cliffe et al., 2014; Foley, Engelhardt & Charles-Dominique, 1995). The seeming contradiction comes from the atypically long digestion period of sloths (McNab, 1982), thought to be the slowest recorded for any herbivorous mammal. For the majority of mammals, digestion rate scales with body size, so larger animals should take longer to digest their food. For sloths, estimations range from 157 h to 50 days (1,200 h) for the passage of food from ingestion to excretion (Foley, Engelhardt & Charles-Dominique, 1995; Montgomery & Sunquist, 1978). In contrast, the food passage time for other similarly sized, foregut fermenters including the Javan langur (*Trachypithecus auratus*, 5–15 kg), red kangaroo (*Macropus rufus*; 18–90 kg), and colobine monkeys (*Colobus angolensis, C. polykomos, Trachypithecus johnii*; 5–20 kg) are just 42, 30, and 54 h respectively (Nijboer et al., 2007; Schwarm et al., 2009). In tandem with a slow rate of digestion, the sloths’ four-chambered stomach is constantly full and so more leaves can only be ingested when digesta leave the stomach and enter the small intestine. This means that food intake and energy expenditure are likely limited by digestion rate and room in the stomach.

Estimations on the exact level of food intake for *Bradypus* sloths vary widely, with predictions ranging between 20 and 60 g dry mass of food per sloth per day (McNab, 1978; Montgomery & Sunquist, 1975; Nagy & Montgomery, 1980). The uncertainty surrounding these values is likely due to the difficulty of following and observing a cryptic arboreal mammal in the wild. Furthermore, *Bradypus* sloths do not adapt well to captivity (Crandall, 1964) and very few institutions are able to maintain a healthy population of *B*. *variegatus*, in large part due to their highly specific and poorly understood diet (Raines, 2005).

Sloths are foregut fermenters, an efficient digestive strategy for low quality food such as leaves, where the microbiome of the stomach is responsible for the break-down of tough plant matter via fermentation (Foley, Engelhardt & Charles-Dominique, 1995). The fermenting microbes are known to function within an optimum temperature range, with maximum productivity occurring at the higher temperatures within this species-specific range (King et al., 2011). Considering that sloths have a low and variable body temperature, it is plausible that ambient temperature will directly affect the fermentation rate of the gut contents in these animals, with higher temperatures resulting in increased fermentation rates. Similarly, the naked mole-rat (*Heterocephalus glaber*) experiences large daily fluctuations in body temperature which has an impact on the caecal fermentation efficiency, with the microbial organisms aiding digestion functioning maximally at the animal’s preferred body temperature of 33 °C (Yahav & Buffenstein, 1991).

We hypothesised that, given the sloth’s constantly full stomach, an increase in fermentation rate should, theoretically, result in an increase in subsequent food intake. If true, this result would provide a link between sloth dietary ecology and their thermoregulatory physiology in a manner that would seem unique amongst mammals. To test this idea, we measured exact levels of food intake in *Bradypus variegatus* sloths over a five month period, to investigate how food intake was affected by natural changes in ambient temperature.

## Material and Methods

### Selection criteria

Three adult female *B. variegatus* sloths (hereafter referred to as *a*, *b*, and *c*) were selected for the study. Females were chosen to reduce confounding effects of male vs female metabolic rates (Selman et al., 2001) given the small sample size. All three animals had been maintained at the Sloth Sanctuary for a minimum of two years prior to the study. The Sloth Sanctuary is a specialist research and care facility that rescues injured and orphaned sloths from throughout Costa Rica. The sloths in this study were aged approximately 3 (*a*), 6 (*b*), and 9 (*c*) years old. *B. variegatus* sloths reach their adult size and sexual maturity around 18–24 months of age (Avey-Arroyo & Gage, 2002) so the study animals would not have had a growth rate-biased metabolism. Although the lifespan of *Bradypus* sloths is unknown, the oldest on record is currently 23, indicating that they are long-lived for mammals their size so that the age differences of the study sloths were minimal and as close to young and mature as could be found. None of the sloths included were pregnant at any point in the study. The sloth body masses were measured at the beginning and end of the study.

### Food consumption

Food intake was measured for 158 continuous days between 20 February 2011 and 28 July 2011. Each sloth was offered 160 g of fresh *Cecropia* leaves twice a day (7 am and 3 pm), and any uneaten food was removed, dried, and weighed in order to determine total food intake. The sloths did not eat the food immediately when provided, but rather ate slowly throughout the course of the day. While it is known that *Cecropia* leaves constitute at least a part of the diet of *B. variegatus* in the wild (Chiarello, 1998), observations made during the continued husbandry of these animals show that *Cecropia* is one of the few plants that all *Bradypus*, regardless of personal preference (Montgomery & Sunquist, 1978), will consume in captivity (Avey-Arroyo & Gage, 2002). Although recent work suggests that wild sloths supplement their diet by consuming algae that seasonally grows on the fur (Pauli et al., 2014), wild sloths in the region were not observed to have algae during the study period, and the animals used in this study did not have algae growing on their fur when entering captivity. Therefore, in order to limit variability, the experimental protocol simulates this condition. To determine the dry weight of the food presented, 10 samples of 160 g of fresh *Cecropia* leaves were dried in a Nesco Gardenmaster^®^ Food Dehydrator (Nesco, Two Falls, Wisconsin, USA) at 32 °C until they reached constant mass. The mean mass of the 10 samples was 60.0 g indicating a water weight of 62.5%, so that 160 g of fresh *Cecropia* leaves could be equated to 60 g dry weight.

### Housing and temperature control

The sloths were housed in individual standardised enclosures measuring 5.3 m^2^ with a shelf measuring 114 cm by 61 cm and 13 horizontal climbing bars. The enclosures were outdoors, so the sloths were subject to natural ambient temperature fluctuations, essentially as if they were in the wild. In order to ensure uniform temperatures and minimise possible microclimate differences between enclosures, all enclosures were sheltered using a metal roof to prevent access of rain or direct sunlight. Although levels of non-visible light such as ultraviolet (UV) were not monitored in this study and may play a role in sloth thermoregulation, the metal roofing should have standardised and minimised these effects. The enclosures used represent the best attempt to control environmental variables without adding undue stress to the sloths. *Bradypus* sloths tend not to do well in captivity (Crandall, 1964; Raines, 2005) but the sloths in this study had maintained their health in these enclosures for 2 years. Therefore, it was decided to avoid introducing any additional stress by manipulating temperature directly (Sikes & Gannon, 2011), as stress could bias food intake more than temperature, blurring the effect in question and possibly harming the animals. Ambient temperatures within the enclosures were recorded to the nearest 0.1 °C at 4 h intervals using a ‘NScessity’ Digital Hygrometer/Thermometer NSHG-02X’ (NScessity, Marlow, Bucks, UK) throughout the study period.

The percentage of days that temperature and food intake shifted in the same direction were calculated for sloths *a*, *b* and *c* individually and as a combined mean. These calculations were made separately for the day to day shifts in minimum, mean, and maximum temperatures. To test whether the temperature shift had a delayed effect on levels of food intake, the same calculations were completed with a +1 day offset for food intake. Finally, to see if larger day to day temperature shifts resulted in more frequent food intake shifts in the same direction, the same calculations were completed using data only from days which had a mean temperature change greater than 0.5 °C and 1 °C. All statistical analyses calculated in R.

## Results

### Descriptive statistics

Ambient temperatures within the experimental enclosures ranged from an overall recorded minimum of 20.8 °C to a maximum of 32.0 °C with an average daily range of 6.2 °C (*N* = 948). The weights of the three sloths at the beginning of the study were 4.3 kg (sloth *a*), 4.2 kg (sloth *b*) and 4.3 kg (sloth *c*), and at the end of the 5 month study period were 4.4 kg (sloth *a*), 4.1 kg (sloth *b*) and 4.5 kg (sloth *c*).

The mean fresh mass of *Cecropia* leaves consumed daily per sloth (*N* = 158) was 196 g (sloth *a*), 192 g (sloth *b*) and 196.3 g (sloth *c*). Using the percent water weight calculated above, this equates to a mean dry-weight food intake for each individual per day of 73.5 g (SD 16.7), 72.0 g (SD 16.9) and 73.6 g (SD 20.2) for sloths *a*, *b* and *c*, respectively. When divided by the mean body mass of the three sloths throughout the study period, this translates to a mass-specific food intake of 17 g dry weight food per kg of sloth per day (SD 4.2). The daily food intake for each sloth was normally distributed (Shapiro–Wilk test for normality using P < 0.05: *p*-value *a* = 0.1347; *p*-value *b* = 0.4245; *p*-value *c* = 0.2146). Of the 18,960 g dry-weight of total food provided throughout the study period, the amount consumed by each sloth was 11,615 g (sloth *a*), 11,381 g (sloth *b*) and 11,628 g (sloth *c*), equating to a consumption of 61.3%, 60.0% and 61.3% of food provided, respectively.

**Figure 1:**
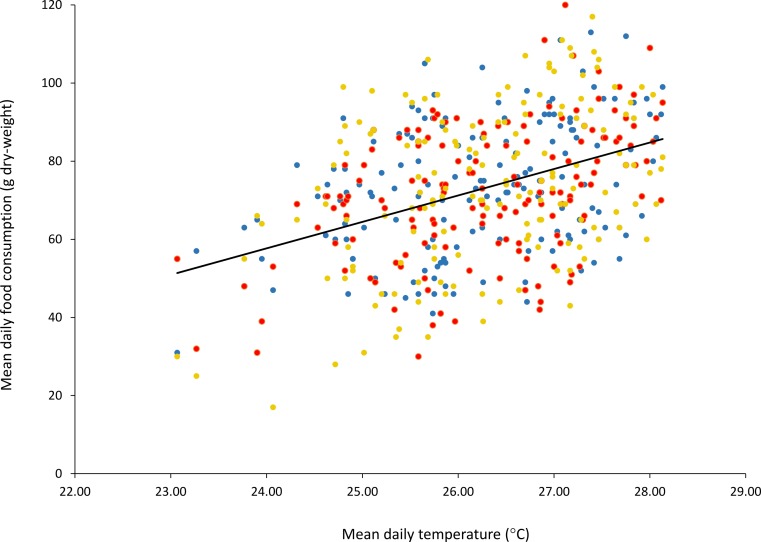
Mean daily food consumption (g dry-weight) for three *B. variegatus* sloths as a function of mean daily ambient temperature (°C). Ambient temperature readings taken at intervals of 4 h for a total of 158 continuous days. Points show means taken over 24 h. Sloth a, blue; sloth b, red; sloth c, yellow.

There was a significant linear relationship between ambient temperature and food intake for all three sloths, with higher food intakes at higher temperatures (sloth *a*: F (1,156) = 25.31, *p* = 1.328e–06, *r*^2^ = 0.14) (sloth *b*: F (1,156) = 38.94, *p* = 3.934*e*–09, *r*2 = 0.20) (sloth *c*: F (1,156) = 30.23, *p* = 1.536e–07, r2 = 0.16) (Fig. 1).

**Figure 2:**
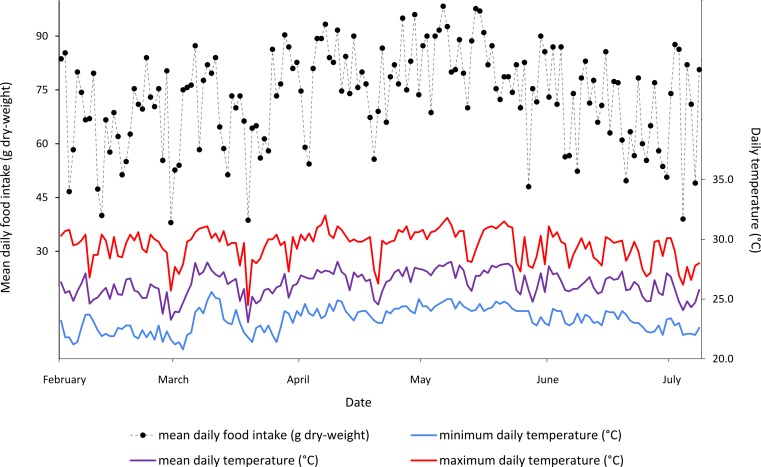
Mean daily food intake (g dry-weight) for three *B. variegatus* sloths and daily temperature (°C) (minimum in blue, mean in purple and maximum in red) throughout the five month study period. Ambient temperature readings taken at intervals of 4 h for a total of 158 continuous days.

In the day-to-day temperature and food intake shift calculations (Table 1), the highest agreement was found between mean daily temperature shifts greater than 1 °C and the mean food intake of all three sloths, with these shifts agreeing on 71.1% of days. In every case, same day comparisons showed higher agreement than one day offset comparisons. In general, the mean daily temperature showed a higher agreement with food intake than either the minimum or the maximum daily temperatures. The results of this analysis can be seen in Table 1 and qualitatively in Fig. 2.

**Table 1:** Percentage of days with a temperature and food intake shift in the same direction. The percentage of shift agreements between temperature and food intake, e.g., if temperature increased, so too did food intake. Table includes minimum temperature shifts, mean temperature shifts, and maximum temperature shifts as compared to daily intake for each sloth individually and the mean intake for all three. Comparisons were made day-to-day and with a one day offset between temperature shift and the next day’s food intake. Finally, the table shows the same calculations excluding days with shifts lower than 0.5 °C and 1.0 °C, respectively.

Daily shift in temperature	Mean of *a*, *b*, & *c*	Sloth *a*	Sloth *b*	Sloth *c*
**Minimum**	50.6%	55.1%	58.2%	57.6%
**Minimum** + **1 day**	46.5%	48.4%	52.9%	52.2%
**Mean**	57.6%	53.8%	54.4%	57.0%
**Mean** + **1 day**	47.1%	46.5%	50.3%	46.5%
**Maximum**	51.3%	52.5%	58.2%	56.3%
**Maximum** + **1 day**	49.7%	48.4%	49.7%	46.5%
**Mean shift >0.5 °C**	65.5%	59.5%	59.9%	58.3%
**Mean shift >0.5 °C** + **1 day**	53.0%	56.6%	50.6%	55.4%
**Mean shift >1 °C**	71.1%	62.2%	66.7%	66.7%
**Mean shift >1 °C** + **1 day**	46.7%	57.8%	53.3%	48.9%

### Comparative statistics

There were no statistically significant differences in the mean food intake of any of the three sloths during the course of the study (Welch’s two sample *t*-test adaptation of the Student’s *t*-test; *a* *vs*. *b*: *p*-value = 0.4341; *a vs. c*: *p*-value = 0.9685; *b vs. c*: *p*-value = 0.4567). As this test compares the mean for the entire period of the study, we also performed a pairwise comparison using a Student’s *t*-test comparing each sloth day-to-day and found no statistically significant differences between any sloth (*a vs. b*: *p*-value = 0.3426; *a vs. c*: *p*-value = 0.9605; *b vs. c*: *p*-value = 0.3615).

However, the daily intake for sloth *c* varied significantly more than for either *a* or *b* (F-test; var *a* = 277.8565, var *b* = 287.0499, var *c* = 408.1151; *p*-values = 0.01651 and 0.02813, respectively) while sloths *a* and *b* did not differ (*p*-value = 0.8387). The complete dataset for this study is available electronically in Table S1.

## Discussion

This study is the first to monitor food intake rates in *Bradypus* sloths for a period appropriate to allow for some estimate of variance. Prior to this study, food consumption estimates for this genus varied widely, probably due to the difficulty of maintaining *Bradypus* sloths in captivity (Crandall, 1964) and the problems of observing a cryptic canopy mammal in the wild. We note that total levels of food intake were remarkably similar for the three animals, with less than a 1.5% difference at the end of the five months. We were able to show that *Bradypus* sloths have an exceptionally low mean food intake of 17 g dry weight kg^−1^ day^−1^, and that this does indeed vary with temperature.

This relationship is evident despite the limitation of a small sample size. By narrowing our sample size, we were able to reduce the variation in our dataset and focus on the potentially causal relationship between temperature and food consumption. Although the addition of more animals may have strengthened the relationship we were investigating, this was not realistic for us given the known difficulty of maintaining healthy *Bradypus* sloths in captivity (Crandall, 1964) under our tightly constrained conditions with respect to age, gender and environment. Even with our small sample size the resultant relationship remains clear, and is manifest at roughly equivalent magnitudes for each animal studied. This suggests that, given the preliminary nature of the work and the consistency of the overall effect, our small sample size controlling for multiple externalities was justified.

The only difference that we found was in the variance of food consumed day-to-day in sloth *c* compared to the other two and this may either be due to individual differences or perhaps an effect of age (sloth *c* was the oldest). Further studies would do well to control temperature directly, but given the limits of maintaining *Bradypus* sloths in captivity, we argue that this work represents an important preliminary step in understanding the dietary habits of *Bradypus* sloths in relation to ambient temperature, setting the stage for future investigations.

While we did not control temperature directly, we did control for the type of food presented to the sloth. Future investigations could vary food type because sloths in the wild are believed to feed from a variety of plant species, although exactly which species and the individual preferences of sloths are poorly understood (Chiarello, 1998; Pauli et al., 2014). It is possible that dietary variation in the wild may affect quantity of food intake due to differences in leaf nutrient content and digestibility but it is difficult to simulate this condition in captivity with enough fidelity to provide a meaningful perspective. Despite this potential complication, we note that our mean food intake rate of 17 g dry weight kg^−1^day^−1^ is very similar to a previous approximation made for wild sloths by Nagy & Montgomery (1980) who, by measuring the increase in concentration of manganese between food consumed and faeces expelled, estimated that an adult *B. variegatus* sloth should consume 60 g dry weight of leaves daily (20 g dry weight kg^−1^day^−1^). This similarity between findings suggests that the wild *Bradypus* diet may be more consistent than previously considered or that leaf variation has minimal impact on overall levels of food intake.

In addition, the concurrence of our values with those of Nagy & Montgomery (1980) suggests that very little difference exists between levels of food intake for wild and captive sloths. This has also recently been observed in several species of primates (Pontzer et al., 2014), and may indicate that levels of energy expenditure for sloths in captivity are similar to their wild counterparts. The low caloric content of leaves obliges most arboreal folivores to consume large quantities of food in order to acquire enough energy (Nagy & Milton, 1979). In contrast, the case of sloths, with a daily food intake of 17 g dry weight kg^−1^day^−1^ is a remarkably low level of consumption for any mammal, and is probably only made possible due to their extremely low metabolic rate as well as behavioural and physiological adaptations that allow them to optimize the use of what few calories they do consume.

Generally, ambient temperature has a direct effect on the metabolic rate and energy expenditure of endothermic mammals (McNab, 2002), with colder temperatures demanding an increased energy requirement due to the thermoregulatory costs of maintaining body temperature (Donhoffer & Vonotzky, 1947). For endotherms that experience extreme seasonal variation in temperature, however, recent studies have shown a surprisingly low level of energy expenditure and food intake during the prolonged cold of winter (Brinkmann et al., 2014; Fletcher et al., 2012; Humphries et al., 2005; Sheriff et al., 2009). This particular response has been attributed to behavioural strategies and physiological adjustments which facilitate a reduction in the energetic requirements to deal with the winter conditions. In the tropics, sloths are not exposed to such prolonged seasonal changes, experiencing only subtle temperature shifts over the yearly cycle. Over shorter timescales though, the decreased level of food intake on colder days by our sloths is at odds with that observed in many other mammals (Brobeck, 1948; Carl & Brown, 1986).

To understand the link between changes in day-to-day temperature and levels of food consumption, we considered how often the two shifts agreed, i.e., moved in the same direction, for all 158 days of the study. In every case calculated, a one day offset yielded less agreement in the shifts than those calculated for the same day. When considering mean food intake for the three sloths, the shifts agreed nearly 58% of the time; although when we only considered shifts in temperature above arbitrary thresholds (0.5 °C and 1 °C) this agreement increased to 65.5% and 71.1%, respectively (Table 1). The greater influence of larger temperature shifts on body temperature would be easy to understand in ectotherms, because a greater differential in temperature would result in a faster rate of heat exchange and we suggest that this is what happened in our sloths (cf. Britton & Atkinson, 1938). Beyond this, a secondary effect is that related to food intake, modulated by gut fermentation rate, itself modulated by temperature. We might expect, however, this trend to be different for wild sloths that have the opportunity to utilise microclimates and bask (Montgomery & Sunquist, 1978), a behaviour which is known to increase their core temperature yet was unavailable to the sloths in this study.

What is then, the precise reason for sloths increasing ingestion rate with increasing temperature? The strong link between sloth core temperature and that of the environment, which has led to them being likened to ectotherms (Britton & Atkinson, 1938), implies that their metabolism will be similarly linked to body, and therefore environmental, temperature (Gillooly et al., 2001). Sloth core temperature will also presumably reflect gut temperature, and this will affect the rate at which the microorganisms in the stomach break down plant matter. In accordance with general microbial metabolic processes (Gillooly et al., 2001), sloth foregut microbes function within an optimum temperature range, with maximum productivity occurring at the higher temperatures within the range observed by us (King et al., 2011). We propose therefore that, in a similar manner to reptiles (Naulleau, 1983), the rate of digestion for *B. variegatus* sloths is likely to be faster and more efficient on days with higher average temperatures. Since sloths maintain a constantly full stomach, they can only ingest more leaves when digesta enter the small intestine, which conforms to our finding of increased food intake on days with higher ambient temperatures. Therefore, if the rate of food passage into the small intestine is causally related to temperature, and temperature was consistent across all three sloths, we should expect that they all would have consumed a similar amount of the food offered to them. In accordance with this hypothesis, we found that the total level of food intake was remarkably similar for each individual (<1.5% difference), with sloths *a* and *c* in particular both consuming 61.3% of food provided over the course of our study.

Although our study is only preliminary, the consistent and significant trend between rate of food consumption and environmental temperature would imply that ambient temperature has profound implications for the rate at which sloths can process the energy they have acquired. This implication, in turn, should affect all aspects of their ecology and physiology that rely upon the expenditure of their consumed energy.

## Conclusions

This study shows that *B. variegatus* sloths have an unusually low mean food intake of 17 g kg^−1^ day^−1^ and have a rate of food intake that is affected by ambient temperature, with increased consumption on hotter days. We propose that, on hotter days, sloth core body temperature increases, increasing the fermentation rate of the gut digesta, enhancing its movement into the small intestine, and allowing for increased food intake to compensate for the emptying stomach. This highlights the significance of a warm, tropical climate in the regulation of metabolic processes in *B. variegatus* sloths. We suggest that maintaining a core body temperature that favours digestive efficiency likely plays a crucial role in balancing a sloth’s daily energy requirements. The adaptive value of behaviours that help modify body temperature that were not controlled for in this study such as basking (Montgomery & Sunquist, 1978) or retraction of the limbs (Dasilva, 1993), now need to be examined to understand better this extraordinary mammal that survives somewhere between poikilothermy and homeothermy. Ultimately, this study demonstrates one of the myriad of ways that mammals are capable of adapting to their environment. *Bradypus* sloths exhibit many evolutionary anomalies relative to the rest of class Mammalia, and a better understanding of their eccentricities paints a fuller picture of the true diversity of mammalian lifestyles and adaptations.

## Supplemental Information

10.7717/peerj.875/supp-1 Supplemental Information 1 Food intake
