# Supplementary material for: Sloths like it hot: ambient temperature modulates food intake in the brown-throated sloth (Bradypus variegatus)
Source: PeerJ. 2015 Apr 2;3:e875. doi: 10.7717/peerj.875 (PMC4389270; doi:10.7717/peerj.875)
Supplement: Supplemental Information 1 [file peerj-03-875-s001.docx]

23.07 31 55 30

23.27 57 32 25

23.77 63 48 55

23.90 65 31 66

23.95 55 39 64

24.07 47 53 17

24.32 79 69 65

24.53 71 63 73

24.62 61 71 69

24.63 74 71 50

24.70 78 68 79

24.72 60 59 28

24.77 70 71 85

24.80 91 69 99

24.82 64 52 50

24.82 78 79 89

24.83 72 66 82

24.83 60 70 65

24.85 46 71 58

24.90 55 60 53

24.90 55 60 52

24.97 74 75 90

25.02 63 79 31

25.08 72 50 87

25.10 71 83 98

25.12 85 88 88

25.13 50 49 43

25.20 77 70 46

25.23 46 68 66

25.33 73 42 46

25.35 65 54 35

25.38 87 86 37

25.40 79 53 54

25.45 45 56 97

25.47 87 88 84

25.52 86 75 95

25.52 94 65 68

25.53 49 63 62

25.58 93 88 76

25.58 46 30 44

25.58 80 84 49

25.58 71 85 85

25.60 72 68 72

25.65 91 75 85

25.65 52 50 68

25.65 105 59 96

25.68 58 47 35

25.68 74 86 106

25.73 91 65 70

25.73 60 93 69

25.73 41 38 70

25.75 46 61 77

25.75 97 91 58

25.75 50 64 55

25.78 53 92 97

25.82 54 41 49

25.83 89 84 90

25.83 70 74 71

25.85 55 73 62

25.85 65 72 62

25.87 91 58 73

25.87 48 90 88

25.87 54 74 46

25.95 46 63 48

25.97 76 39 85

25.98 58 91 84

26.00 84 80 56

26.12 81 52 78

26.12 70 77 83

26.15 86 68 89

26.15 62 77 71

26.18 73 80 82

26.23 75 90 70

26.25 86 64 79

26.25 63 73 70

26.25 104 69 46

26.26 49 66 39

26.27 75 87 86

26.30 71 84 68

26.42 70 89 90

26.42 95 59 97

26.43 60 66 44

26.43 79 50 69

26.48 91 68 90

26.50 85 60 75

26.50 72 84 74

26.52 72 90 99

26.58 74 76 81

26.60 82 67 87

26.62 76 74 82

26.63 59 59 47

26.63 74 57 71

26.68 73 89 95

26.70 49 69 92

26.70 77 47 107

26.72 44 55 64

26.72 98 85 60

26.73 57 70 61

26.75 78 92 72

26.83 71 48 58

26.85 90 72 65

26.85 75 42 97

26.86 75 44 70

26.87 60 69 65

26.87 72 75 75

26.90 92 111 82

26.95 95 94 104

26.95 92 94 105

26.98 96 81 76

26.98 85 66 73

26.98 57 72 77

27.00 92 53 103

27.03 62 61 52

27.07 111 72 69

27.07 89 59 92

27.08 68 69 111

27.08 76 91 94

27.12 82 120 69

27.15 61 80 59

27.17 90 71 52

27.17 91 70 109

27.17 60 49 43

27.18 88 51 107

27.20 88 107 73

27.23 86 93 91

27.23 91 76 79

27.27 65 53 58

27.28 52 85 96

27.28 85 74 65

27.30 103 65 102

27.32 74 89 89

27.32 62 66 55

27.38 113 74 93

27.40 68 88 117

27.42 99 77 63

27.42 54 77 108

27.45 84 80 104

27.47 67 103 60

27.47 60 96 106

27.52 96 86 88

27.53 63 86 72

27.63 96 93 99

27.65 74 85 67

27.68 63 99 63

27.68 55 86 82

27.75 61 79 79

27.75 112 91 92

27.80 83 84 95

27.83 95 89 91

27.83 91 97 79

27.85 69 79 69

27.92 66 71 99

27.97 96 80 60

28.00 92 109 77

28.03 80 85 97

28.07 86 91 69

28.12 92 70 78

28.13 99 95 81
